# Accuracy and Calibration of Computational Approaches for Inpatient Mortality Predictive Modeling

**DOI:** 10.1371/journal.pone.0159046

**Published:** 2016-07-14

**Authors:** Christos T. Nakas, Narayan Schütz, Marcus Werners, Alexander B. Leichtle

**Affiliations:** 1 University Institute of Clinical Chemistry, Centre of Laboratory Medicine, Inselspital, Bern University Hospital, University of Bern, Bern, Switzerland; 2 Laboratory of Biometry, University of Thessaly, Volos, Greece; 3 Central Controlling Unit, Inselspital, Bern University Hospital, University of Bern, Bern, Switzerland; Yokohama City University, JAPAN

## Abstract

Electronic Health Record (EHR) data can be a key resource for decision-making support in clinical practice in the “big data” era. The complete database from early 2012 to late 2015 involving hospital admissions to Inselspital Bern, the largest Swiss University Hospital, was used in this study, involving over 100,000 admissions. Age, sex, and initial laboratory test results were the features/variables of interest for each admission, the outcome being inpatient mortality. Computational decision support systems were utilized for the calculation of the risk of inpatient mortality. We assessed the recently proposed Acute Laboratory Risk of Mortality Score (ALaRMS) model, and further built generalized linear models, generalized estimating equations, artificial neural networks, and decision tree systems for the predictive modeling of the risk of inpatient mortality. The Area Under the ROC Curve (AUC) for ALaRMS marginally corresponded to the anticipated accuracy (AUC = 0.858). Penalized logistic regression methodology provided a better result (AUC = 0.872). Decision tree and neural network-based methodology provided even higher predictive performance (up to AUC = 0.912 and 0.906, respectively). Additionally, decision tree-based methods can efficiently handle Electronic Health Record (EHR) data that have a significant amount of missing records (in up to >50% of the studied features) eliminating the need for imputation in order to have complete data. In conclusion, we show that statistical learning methodology can provide superior predictive performance in comparison to existing methods and can also be production ready. Statistical modeling procedures provided unbiased, well-calibrated models that can be efficient decision support tools for predicting inpatient mortality and assigning preventive measures.

## Introduction

The use of Electronic Health Records (EHR) for building mortality predictive models is a modern practice that is expected to enhance patient care by pointing physicians to patients at risk, that would potentially be missed in clinical routine [1]. The vast amount of EHR and laboratory databases allow for the construction of scoring models that quantify patients’ risks for event or mortality prediction and strongly suggest the use of emerging “big data”strategies for analyzing these frequently incomplete, unordered, and usually for documentation purposes only, collected data [2–4].

Models such as the Acute Laboratory Risk of Mortality (ALaRMS) score [5,6] have been proposed in the literature to this goal, as the rapid assessment of clinical severity using EHR data available at the time of admission may aid decision support and improve healthcare quality. For an elderly cohort in Israel, Smolin et al. [7] applied multivariate logistic regression analysis to predict the risk of 6-month mortality from laboratory and clinical anamnesis data, whereas Lee et al. [8] proposed a method for personalized mortality prediction based on electronic health record (EHR) data and patient similarity metrics. Scoring systems such as the Acute Physiology and Chronic Health Evaluation (APACHE IV), the Simplified Acute Physiology Score (SAPS), the Laboratory-based Acute Physiology Score (LAPS), the COmorbidity Point Score (COPS), and others are typically used in clinical practice and may aid to the decision making process offering a simplified, swift patient assessment [2, 3]. However, such scoring systems almost always offer simplistic quantification of patients’ risks.

In this work, we reproduced the method proposed by Tabak et al. [5], namely the Acute Laboratory Risk of Mortality Score (ALaRMS) as a modern representative of scoring systems, built our models using statistical learning methodology, and compared all of them. We show that models that are constructed based on statistical learning methodology are well-calibrated and offer superior diagnostic accuracy to previous or traditional regression approaches.

As a result, statistical learning methodologies may provide more accurate predictions and enable the use of incomplete data, not only for retrospective studies but potentially also for real-time application.

The following section describes the database on which we based our assessment, develops on the methods we used, and covers the implementation procedures. Results are presented next along with intuition about these. We end with a discussion and conclusions about proper strategies for predicting inpatient mortality based on relevant retrospective outcome studies.

## Methods

In this section, we present the experiment design which simply involves the extraction of the database and methodology used for modeling purposes.

### Database

The complete database of hospital admissions to the Inselspital from early 2012 to late 2015 was used, involving over 100,000 admissions. The Inselspital provides as the University Hospital of Bern quaternary medical care mainly for the canton of Bern (about one million inhabitants) with 78 departments, about 900 beds, and more than 6000 employees (17% physicians, 38% care-givers). Age, sex, and initial laboratory test results were included in the database, the outcome being discharge disposition, which identifies inpatient mortality status. A total of 23 numeric laboratory test results were included for reasons of consistency with the construction of the ALaRMS model. Those were serum chemistry (specifically: albumin, aspartate trans-aminase, alkaline phosphatase, blood urea nitrogen (BUN), calcium, creatinine, glucose, potassium (K), sodium (Na), and total bilirubin); hematology and coagulation parameters (bands, hemoglobin, partial thromboplastin time, prothrombin time international normalized ratio (PT INR), platelets, and white blood cell count (WBC)); arterial blood gas (partial pressure of carbon dioxide (pCO_2_), partial pressure of oxygen (pO_2_), and pH value); cardiac markers (brain natriuretic peptide (BNP) or NT-proBNP, creatine phosphokinase MB (CK MB), and troponin T (to replace troponin I in the score). An ethics dispensation from the cantonal ethics committee Bern (№Z023/2014) was issued for the anonymized use of these data.

Data handling involved filtering and pre-processing. Entries with missing values for discharge disposition and patient/admission ID were eliminated. A single duplicate entry was found and verified which was also eliminated from the database. The final database included 106,688 admissions. Crude inpatient mortality risk was calculated to be 2.41% (corresponding to 2,568 admissions). Multiple imputation was used as a second step in order to handle missing data since not all patients had results for the 23 laboratory tests that were considered. We have then employed modeling approaches both for the database with the missing and for the one with the imputed data.

### Statistical analysis methods and implementation

Initial data analysis involved the calculation of basic descriptive statistics based on the available data as a first step. Next, the ALaRMS score was calculated for each admission along the lines described in Tabak et al. [5]. The ALaRMS system was chosen as representative of scoring systems being a most recent addition to the relevant bibliography [5]. The following step was to build statistical models that can be used as a prognostic tool for inpatient mortality combining age, sex, and lab measurements per se, without having to resort to a scoring system. A random 80% of the admissions were used as training sample while the rest were used as the testing sample.

Linear classification methodology included generalized estimating equation (GEE) techniques and Generalized Linear Modeling using LASSO (GLM). Regarding the GEE model, admission ID defined the subject-level and age, sex, and the different laboratory test measurements were introduced as explanatory variables. GEEs can handle missing values effectively and were thus used for the non-imputed database. GEE parameter estimates are consistent even when the covariance structure is misspecified [9]. We have used an independent structure for the working correlation matrix, which reduced significantly computational burden.

GLM was considered for an imputed dataset. Specifically, we have used penalized multiple logistic regression with LASSO penalization, a choice that can be equivalent to Bayesian Model Averaging [10]. The tuning parameter lambda was chosen via cross-validation [11].

Neural artificial network and decision tree based algorithms were adjusted to be directly usable on raw data. For the neural network we employed a Multi-Layer Perceptron (MLP) with 3 hidden layers, rectified linear units (as activation function for the hidden units) and dropout (for better generalization).

For the decision tree approach we used a rule-based model via the C5.0 algorithm [12]. This algorithm was chosen as it is able to handle missing data relatively well, and besides computational performance gains, also allows for boosting out of the box, which generally results in better predictive performance. In the final decision rule model, an ensemble of 11 models was used for prediction. Soft-classification was used for both the MLP and C5 model, in order to derive probabilistic predictions for class assignment. For calibration a simple logistic regression was put on top of both models to calculate the respective class posterior probabilities. ROC curve analysis was used for the assessment of the accuracy of markers and scores that were calculated.

The R software [13] was used for data pre-processing (packages used: ‘dplyr’ [14], ‘plyr’ [15]], ‘tidyr’ [16], ‘doBy’ [17]) and data analysis (packages used: ‘lme4’ [18] for the GEE, ‘pROC’ [19] for ROC analysis, ‘glmnet’ [20] for binary multiple logistic regression with LASSO penalization, ‘C50’ [21] for the application of the C5.0 algorithm for the rule-based model, ‘RSNNS’ [22] for the MLP). Multiple imputation was applied using the ‘mi’ package [23]. Additionally, Stata 13.1 (Stata Corp., College Station, TX) was used for the calculation of descriptive statistics, linear regression modeling, generalized estimating equation modeling verification, and figure art.

## Results

For the admissions to Inselspital Bern recorded between 2012 and 2015, a complete database was built including information such as age, sex, and initial laboratory test results along with the outcome, discharged ‘alive’ or ‘dead’. After data cleaning 106,688 records remained for analysis. There were 48,497 women (45.5%) and 58,191 men (54.5%) with 1,030 deaths among women (2.1%) and 1,538 deaths among men (2.6%). The average age was 52.03 (±24.66), being 51.69 (±24.68) for those discharged alive and 66.16 (±19.42) for those discharged dead.

The average number of initial laboratory tests administered was 10.58 (±4.48). Among those who died the average number of administered tests were 15.11 (±4.03), while they were equal to 10.46 (±4.43) among those discharged alive. The average ALaRMS score was 16.98 (±18.50), being 16.21 (±17.59) for those discharged alive and 48.50 (±25.38) for those discharged dead.

Table 1 shows descriptive statistics for laboratory test results for all 106,688 admissions.

**Table 1:** Laboratory test results descriptive statistics for all admissions.

Test	Mean	SD	Median	min	max
ASAT (U/L)	61.67	345.9	26	4	26442
Albumin (g/L)	31.16	7.03	32	4	56
BNP (pg/mL)	631.35	898.57	260	10	5000
(NT-pro)-BNP (pg/mL)	5196.21	13385.65	1484	5	299792
CK-MB (μg/L)	8.98	30.63	2	0.3	396.7
Calcium (mmol/L)	2.23	0.2	2	0.5	15.47
Creatinin (μmol/L)	89.8	82.21	73	3	3334
Glucose (mmol/L)	6.67	3.12	5.9	0.2	101
Urea (mmol/L)	7.82	6.23	6	0.8	98.4
Hemoglobin (g/L)	127.65	20.46	130	1	238
INR	1.18	0.52	1.02	1	11
Potassium (mmol/L)	4.08	0.6	4	1.5	30.9
Leukocytes (G/L)	9.02	7.03	8	0	575
Sodium (mmol/L)	138.7	3.8	139	65	182
Band neutr. (%)	16.9	14.21	13.5	0	91.5
Thrombocytes (G/L)	238.35	97.31	225	1	2848
Troponin T (μg/L)	0.28	1.43	0.021	0.003	84.4
aPTT (s)	40.53	36.8	31.5	0	300
alk. Phosphatase (U/L)	106.56	130.52	74	10	8156
pCO_2_ (mmHg)	40.7	9.99	39	7	172
pH	7.38	0.089	7.39	5.85	7.79
pO_2_ (mmHg)	98.62	91.95	66	0	3678

**Fig 1:**
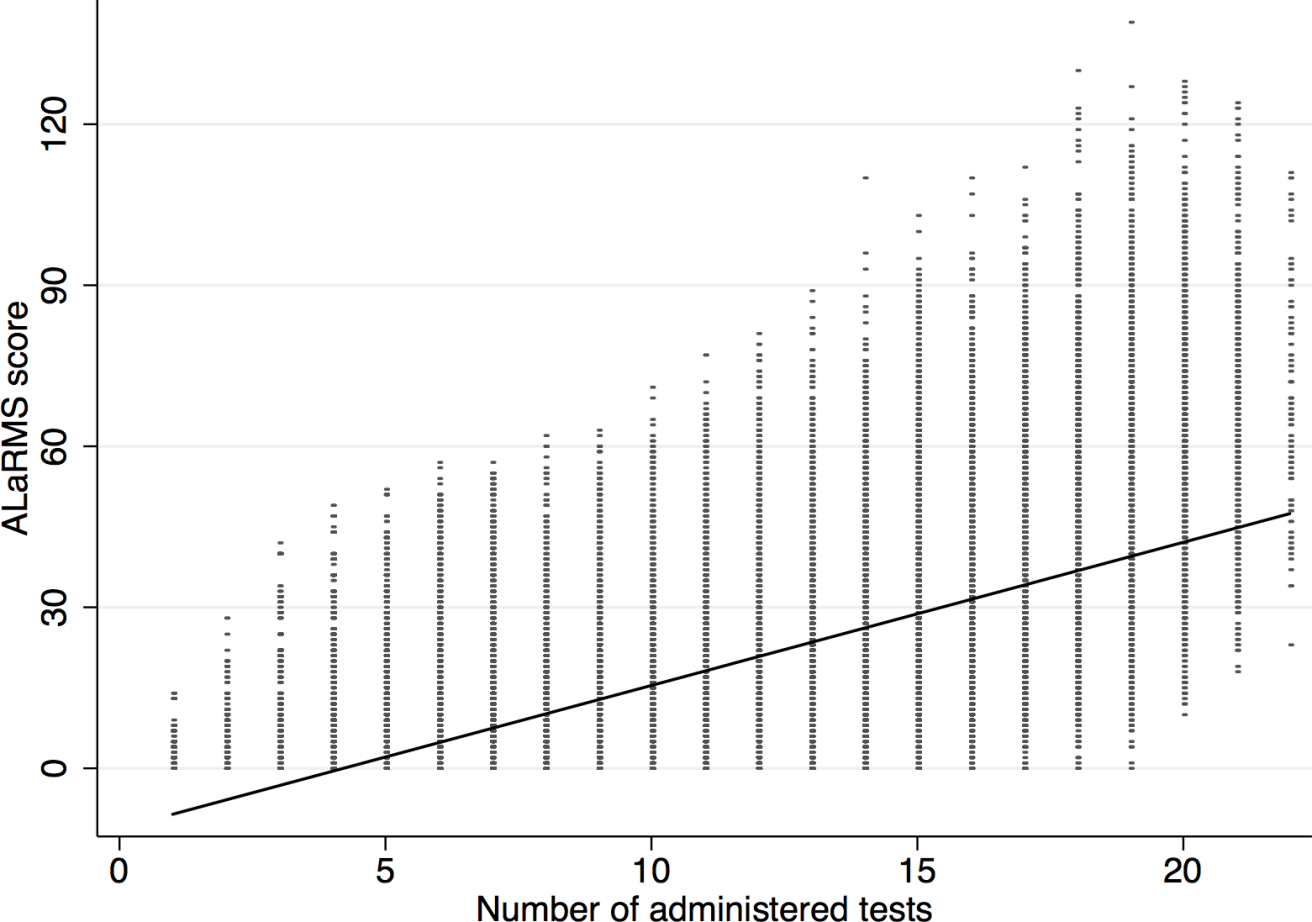
Linear trend of ALaRMS score vs. number of administered tests ordered by the physician in charge.

The ALaRMS score has been proposed as a tool for the prediction of death based on age, sex, and initial laboratory test results [5]. Our results regarding the accuracy of ALaRMS resemble those reported in [5,24,25]. Specifically, the area under the ROC curve was estimated to be equal to 0.858 (95% CI: 0.851, 0.865) comparable to 0.87 in the publication introducing this [5]. However, our findings demonstrate a clear linear trend between the ALaRMS score and the number of tests ordered by the physicians [26] (cf. Fig 1). We eliminated this trend using the ratio of the ALaRMS score over the number of administered tests as a prognostic index for death. As expected, the resulting AUC for the ratio had lower accuracy, being equal to 0.819 (95%CI: 0.813,0.826) and comparable to the accuracy of a model that simply combines the number of administered tests plus age and sex (model shown in Table 2). Simply using the number of administered tests to predict death results in AUC equal to 0.786 (95% CI: 0.777, 0.795), while adding age and sex as predictors to a binary logistic regression model yields AUC equal to 0.801 (95% CI: 0.792, 0.809).

**Table 2:** A simplistic alternative to the ALaRMS score with comparable accuracy to the adjusted-for-number of administered tests ALaRMS score.

Parameter	B	Std. Error	Sig.	OR	95% CI for OR	
(Intercept)	-7.941	0.1093	<0.01	-		
Sex = F	-0.034	0.0416	0.415	0.967	0.891	1.049
Number of administered tests	0.232	0.0054	<0.01	1.262	1.248	1.275
Age	0.021	0.0012	<0.01	1.021	1.018	1.023

Furthermore, considering the fact that the prior probability of death is quite low (2.41%), the positive predictive value (PPV) of the ALaRMS model is significantly weakened. For example, the pair of sensitivity and specificity corresponding to the Youden index-based cut-off point for the ALaRMS model was estimated to be 81.1% and 74.4% respectively. These result in a PPV = 7.26% and a high negative predictive value (NPV) equal to 99.38%. In order to achieve sensitivity in the vicinity of 80% one needs to select a cut-off point of 25 for the ALaRMS score. A cut-off point equal to 73 would result in a much higher PPV (i.e. PPV = 30.53% with corresponding NPV = 98%), but in this case the sensitivity of ALaRMS drops to 18%.

**Fig 2:**
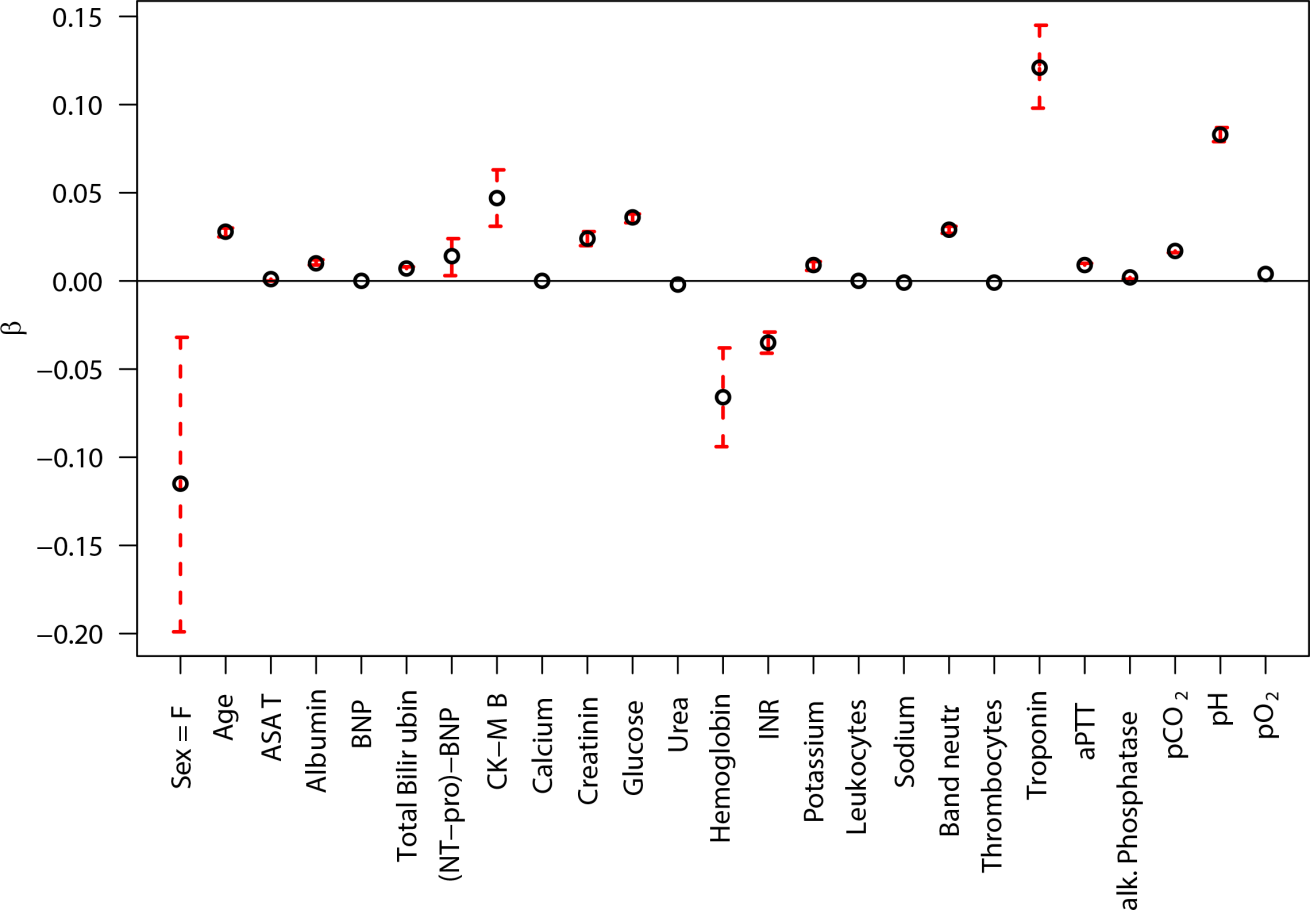
Absolute values of model parameters, along with 95% confidence intervals, for the GEE model.

The resulting AUC for the GEE for the testing sample was 0.809 (95% CI: 0.801, 0.817). A graphical representation of the coefficients of the GEE model is shown in Fig 2. Although the importance of specific laboratory measurements on the outcome is obvious and may provide clinical insight, this model does not provide an adequate accuracy in terms of ROC AUC. This model was applied to the non-imputed dataset, as it can easily handle missing data. However, application of the GEE to the imputed dataset resulted in a very slight increase of the ROC AUC (0.814).

**Fig 3:**
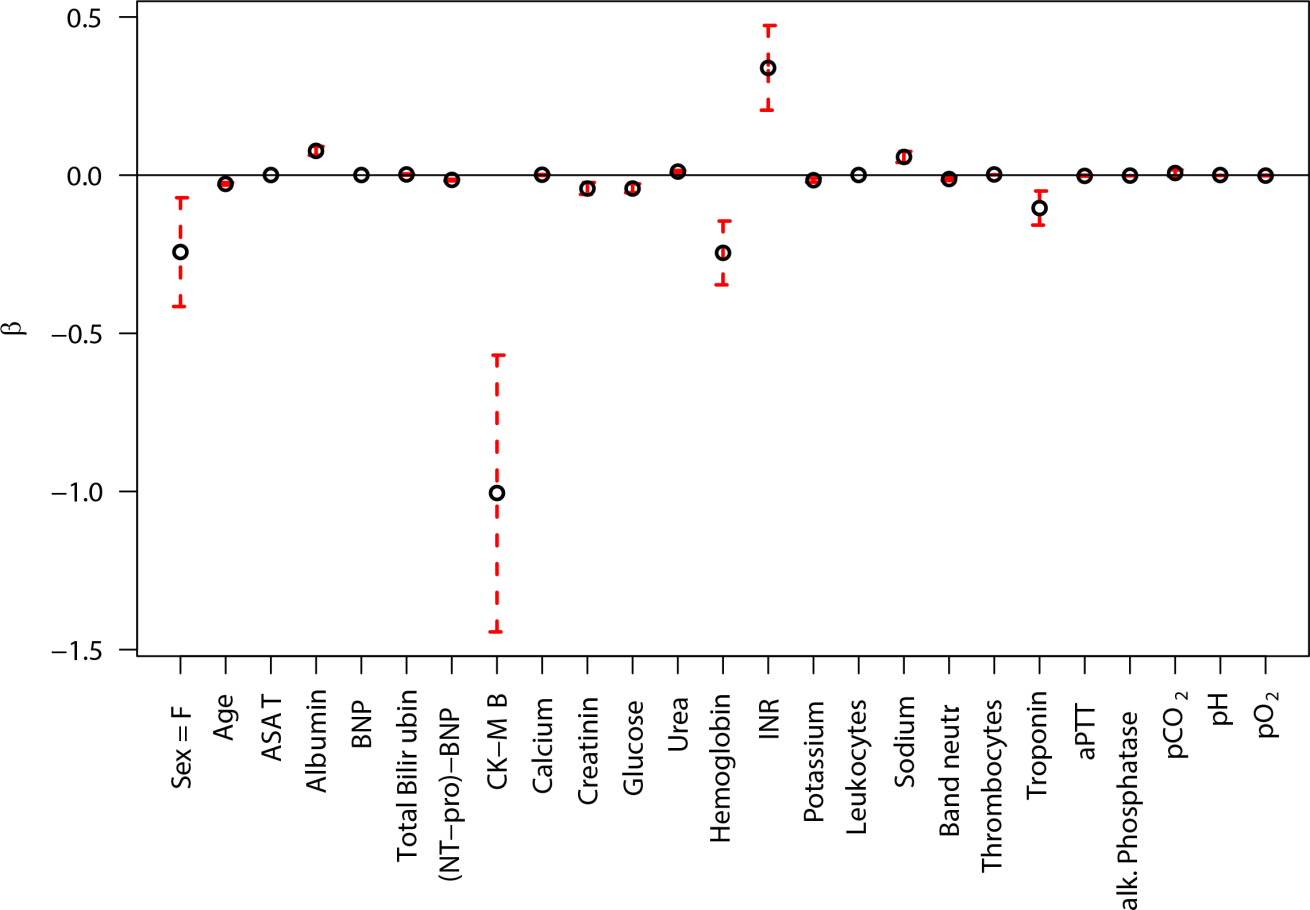
Absolute values of model parameters, along with 95% confidence intervals, for the GLM.

Model parameters after imputation and restricted multiple logistic regression with LASSO penalization, which yielded an AUC equal to 0.872 (95% CI: 0.859, 0.885) for the testing sample, are presented in Fig 3.

**Fig 4:**
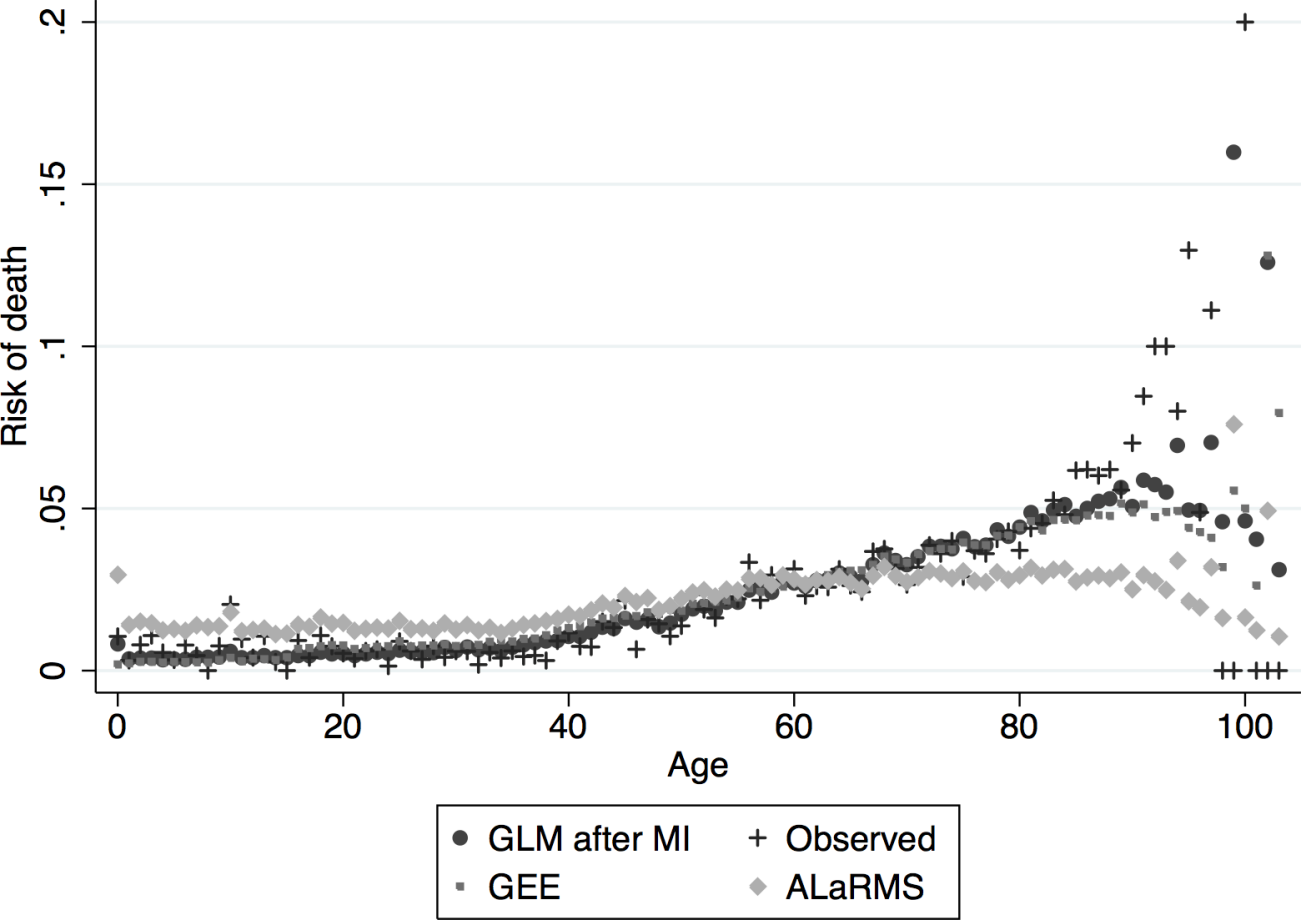
Model calibration according to ‘Age’. Observed vs. Expected risk of death for GEE, GLM, and ALaRMS.

**Fig 5:**
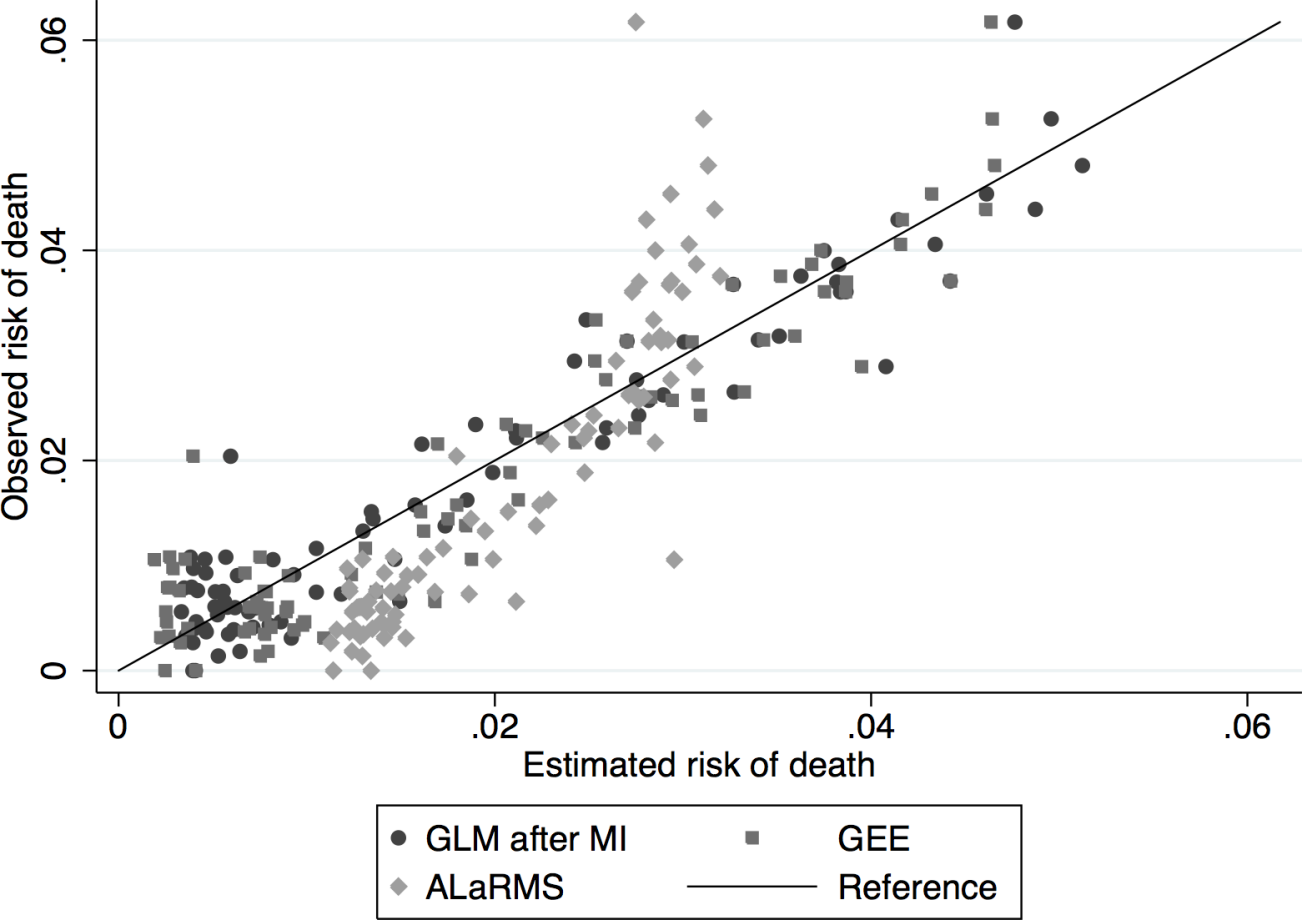
Model calibration according to ‘Age’. Expected vs. Observed risk of death for GEE, GLM, ALaRMS.

Similar results, in terms of accuracy, were obtained for the rule-based random forest model (C5.0) and MLP. Specifically, C5.0 using soft classification, without feature selection on the imputed data resulted in AUC equal to 0.870 (95% CI: 0.862, 0.878) for the testing sample. The model using feature selection resulted in ROC AUC of 0.847 (95% CI: 0.834, 0.857). Attribute importance of the C5.0 model is shown in Table 3, the more important a variable in the model is, the more important it is for making decisions regarding class assignment, thus the higher its discriminative power is. However, given that these classification models are not naturally probabilistic, they may produce distorted class probability distributions [27]. Calibration of these models was achieved using standard logistic regression after initial soft-classification, with this methodology we can directly calculate the respective class posterior probabilities which results in well calibrated models. The fit and model calibration of the ALaRMS, GEE, and GLM relative to patients’ age are shown in Figs 4 and 5.

**Table 3:** Attribute importance (average of 11 decision trees; C5.0 algorithms used splits) for the imputed dataset.

100.00%	Band neutr.		
100.00%	pH	98.12%	CK-MB
100.00%	Calcium	97.96%	BNP
99.94%	ASAT	97.67%	(NT-pro) BNP
99.92%	Hemoglobin	96.69%	Thrombocytes
99.90%	pCO_2_	96.54%	Albumin
99.59%	Age	95.20%	Creatinin
99.28%	pO_2_	95.18%	Glucose
99.15%	Urea	86.10%	Sodium
99.02%	Total Bilirubin	67.07%	INR
98.49%	Leukocytes	44.73%	Sex
98.35%	alk. Phosphatase	32.17%	Troponin T
98.24%	aPTT	0.42%	Potassium

## Discussion

We demonstrated that statistical learning methodology could provide superior predictive performance in comparison to existing methods given that estimated ROC AUCs of the models that were built provide evidence of better prognostic accuracy. The ALaRMS score that has been recently proposed as an indicator signaling inpatient mortality for a hospital admission would in our case constitute a biased assessment largely influenced by the subjective opinion of the physician ordering a batch of lab tests for the admitted patient [28] as, by its methodological construction, it takes on larger values as the number of administered tests increases. The ALaRMS score also suffers from poor calibration based on our findings. Counting the number of tests requested for a certain patient and combining it with age and sex yields an AUC of 0.801 (compared to 0.858 of the ALaRMS score). Since ALaRMS is an additive score of points assigned to a limited number of lab result ranges, it only roughly covers continuous effects and does not account for e.g. mutual information. However, innovative predictive modeling strategies provide sufficient accuracy in incomplete, biased data sets like EHR and laboratory data [2–4].

Imputation allows building a model as if no biased assessment existed. Our results show that this works pretty well in practice. A possible drawback is the large amount of missing values and that the GLM results in high accuracy when we actually use imputation. However, using modern algorithms for boosting the performance of rule-based methods, one can produce models with high accuracy without any imputation, since we have achieved AUCs of over 0.90 (specifically, up to AUC = 0.912 and 0.906, for the C5 and MLP respectively) by properly adjusting the rule-based algorithms parameters (feature weighing) and sampling schemes (subject selection).

Although lab requests usually should follow uniform diagnostic paths, their patterns are frequently highly variable–between disciplines, between clinics, and between physicians. Open “menu” request systems without digital expert systems or administrative restrictions facilitate the selection of favorite sets of “biomarkers”, that are neither mirrored by the actual guidelines nor by computational evidence [29], and even if published or hospital-based recommendations exist, they are scarcely followed [30]. The high degree of collinearity present in many routinely measured lab results not only points at tests potentially not additionally informative, but also blurs the contribution of a single parameter to a certain prediction or differentiation. Statistical learning approaches such as in our case the MLP and C5.0 algorithm can incorporate not only collinearities but also mutual information between individual variables.

Albumin e.g. as a negative acute-phase protein is inversely correlated to markers of inflammation (e.g. leukocyte count), and CK-MB and Troponin as markers of myocardial damage are frequently jointly elevated. The actual “diagnostic value” of a given lab test therefore depends not only from its actual level, but also from its shared variance with other markers and also the pre-test probability in the respective patient population. The assessment of a lab test’s predictivity for a certain end point or differential diagnosis therefore remains a medical as well as computational challenge with great implications on patient care and healthcare costs.

Comparison of the GEE model coefficients in Fig 2 with the GLM coefficients in Fig 3 suggests that low hemoglobin is significant for the GEE model (typically associated with e.g. kidney problems, GI bleeding, and trauma), whereas high hemoglobin values are important in the GLM model (associated especially with bone marrow disorders). Although smaller in magnitude, there appears to be also a reversal in estimated influence of albumin. This suggests that separate modeling of elevated versus depressed levels for some markers may improve model performance. We can also assume that within the whole “model space” there are many opposing and inverted, but likewise predictive marker combinations. A second observation in looking at the model fits is the large coefficient for Troponin in the GEE model with smaller importance of CK-MB, with the GLM model having a very large coefficient for CK-MB and small for Troponin. Given that data is missing on occasion, a model approach that puts similar weights on highly correlated factors could improve model predictiveness for individuals where one or the other measurement is missing. Feature/marker weighing and subject selection are automatically accommodated with the C5 and MLP approaches but not with the current GLM and GEE ones. Future work includes further refinement of all of our models and production of a ready-to-use system for everyday clinical practice.

## Conclusion

Employing computation intensive methods for decision support could help save patients’ lives as they may help physicians to better assess a patient’s risk. Efficient strategies that link machine learning methodology to clinical decision making are described in this article and could enhance diagnostic accuracy and patient assessment.

Future research also includes expanding such models to more types of data, for example, medication, clinical history, proteomics/metabolomics data, microarray data or even full genome scans.
